# 2024 Acknowledgment of *Microbiology Spectrum* Reviewing Editors

**DOI:** 10.1128/spectrum.00404-24

**Published:** 2024-04-02

**Authors:** Christina A. Cuomo

**Affiliations:** 1 Brown University, Providence, Rhode Island, USA

## EDITORIAL

The Reviewing Editor program at *Microbiology Spectrum* is open to early-career microbiologists from across the globe. The Reviewing Editor role is distinct from the Editor role in that while Editors choose peer reviewers and make decisions on papers, the Reviewing Editors serve as expert reviewers for one to two papers per month (a maximum of 24 per year) and receive feedback on their reviews. Reviewing Editors advance to the Editor role on the basis of their performance and subject area expertise needs for Editors.

Currently, we have 94 Reviewing Editors from 28 countries with expertise across microbiology. In the past year, our Reviewing Editors provided over 700 reviews for the journal—extraordinary service for which we are very grateful. Furthermore, over one-third of every manuscript reviewed was evaluated by at least one Reviewing Editor.

This past year, we promoted 20 Reviewing Editors to the Editor role. These individuals were selected based on their subject area expertise, number of reviews completed, and the quality of the reviews. Our plan is to continue to promote Reviewing Editors every 6 months.

We thank all of our Reviewing Editors, past and present, for being a part of this new and evolving program. Please join us in congratulating the following people who served in the Reviewing Editor role in 2023 (Reviewing Editors who were promoted to the Editor role are bolded):

Philips Akinwole

**Valeria Allizond**

Cassio Almeida-da-Silva

Megan Amerson-Brown

Jorge Arca-Suárez

Charina Gracia Banaay

Arden Baylink

Michael Becker

Miguel Angel Garcia Bereguiain

Irene Burckhardt

**Ana Cabrera**

Amanda Calvert

Cecilia Carvalhaes

Alexandra Chiaverini

**Siu-Kei (Jacky) Chow**

Vito Colella

Francesco Comandatore

**Bernadette Connors**

Jôice Corrêa

**Luciana Costa**

Sneha Couvillion

Maria De Francesco

Kanan Desai

Bismarck Dinko

Alex Dulovic

Erin Eggleston

**Yi-Chin Fan**

Jia-Yih Feng

Madiha Fida

Carlos Figueroa Castro

Hui-Chen Foreman

Estela Galvan

Ketaki Ganti

**Sophia Georghiou**

Emilia Ghelardi

Shannon Hinsa-Leasure

Laura Huber

Yosef Huberman

Kelly Ishida

Juan Jaworski

Teny John

Hyung Jong Jin

Lovleen Joshi

Denis E. Kainov

Jaspreet Kaur

Johanna Kenyon

**Katharina Kujala**

Wentao Li

Yongxin Li

Xiaoyan Lin

Amelia Lindsey

**Benjamin Liu**

Hao-Yu Liu

Veranja Liyanapathirana

Sonia Luque

**Zhe Lyu**

**Jose Martinez-Navio**

Patrick McDaneld

Jun Miao

Sheema Mir

Sarita Mohapatra

Walaa Mousa

Shahzad Munir

Azdayanti Muslim

**Dhammika Navarathna**

Jowita Samanta Niczyporuk

**Kattia Núñez-Montero**

Michael Owusu

Andrea Prinzi

Elias Rahal

Ashok Reddy

**Kessendri Reddy**

Christopher Rice

**Antonio Ruzzini**

Sanghamitra Saha

Jennifer Salerno

Srujana Samhita Yadavalli

Garima Singh

Nicolas Soler

Jaime Soria

Katie Summers

Hao Tan

Fang Tang

Li Tao

Anuraj Theradiyil Sukumaran

**Cecilia Thompson**

Sangeeta Tiwari

**Felix Toka**

Maria Tomas

Xinzhao Tong

Deeksha Tripathi

Gareth Trubl

Pablo Tsukayama

Filipa Vale

Vanessa Varaljay

Musturi Venkataramana

Crista Wadsworth

Caray Walker

Fang Wang

Wei Wang

Yan Wang

Yi Wang

**Bobby Warren**

Michael Whitfield

Natalie Whitfield

**Gregory Wiedman**

**Anne Wyllie**

Zhu Xiaoyu

Yifei Xu

Pragya Yadav

Rebecca Yee

Jinki Yeom

Kayvan Zainabadi

Krzysztof Zawierucha

Jianhua Zhang

Xue Zhang

